# Comprehensive Analysis of Tissue Preservation and Recording Quality from Chronic Multielectrode Implants

**DOI:** 10.1371/journal.pone.0027554

**Published:** 2011-11-09

**Authors:** Marco Aurelio M. Freire, Edgard Morya, Jean Faber, Jose Ronaldo Santos, Joanilson S. Guimaraes, Nelson A. M. Lemos, Koichi Sameshima, Antonio Pereira, Sidarta Ribeiro, Miguel A. L. Nicolelis

**Affiliations:** 1 Edmond and Lily Safra International Institute of Neuroscience of Natal, Natal/RN, Brazil; 2 Clinical Neurophysiology Laboratory of the Associação Alberto Santos Dumont para Apoio a Pesquisa, Sírio Libanês Hospital, São Paulo/SP, Brazil; 3 Foundation Nanosciences and Clinatec/LETI/CEA, Grenoble, France; 4 Department of Radiology, School of Medicine, University of São Paulo, São Paulo/SP, Brazil; 5 Brain Institute, Federal University of Rio Grande do Norte, Natal, RN, Brazil; 6 Center for Neuroengineering, Department of Neurobiology, Duke University Medical Center, Durham, North Carolina, United States of America; 7 Department of Biomedical Engineering, Duke University, Durham, North Carolina, United States of America; 8 Department of Psychological and Brain Sciences, Duke University, Durham, North Carolina, United States of America; Nathan Kline Institute and New York University School of Medicine, United States of America

## Abstract

Multielectrodes have been used with great success to simultaneously record the activity of neuronal populations in awake, behaving animals. In particular, there is great promise in the use of this technique to allow the control of neuroprosthetic devices by human patients. However, it is crucial to fully characterize the tissue response to the chronic implants in animal models ahead of the initiation of human clinical trials. Here we evaluated the effects of unilateral multielectrode implants on the motor cortex of rats weekly recorded for 1–6 months using several histological methods to assess metabolic markers, inflammatory response, immediate-early gene (IEG) expression, cytoskeletal integrity and apoptotic profiles. We also investigated the correlations between each of these features and firing rates, to estimate the impact of post-implant time on neuronal recordings. Overall, limited neuronal loss and glial activation were observed on the implanted sites. Reactivity to enzymatic metabolic markers and IEG expression were not significantly different between implanted and non-implanted hemispheres. Multielectrode recordings remained viable for up to 6 months after implantation, and firing rates correlated well to the histochemical and immunohistochemical markers. Altogether, our results indicate that chronic tungsten multielectrode implants do not substantially alter the histological and functional integrity of target sites in the cerebral cortex.

## Introduction

The use of chronic multielectrodes for recording the activity of neuronal populations in awake behaving animals [1]–[6] represented a great step forward in our understanding of the function of the brain. In particular, there is great promise in the use of this approach to allow human patients to control neuroprosthetic devices, designed to restore function after either traumatic injury of the central nervous system (CNS) or neurodegenerative diseases [7]–[14].

An important prerequisite for any invasive brain machine interface is to maintain a stable signal in the CNS for the longest time possible without causing either structural, cellular or metabolic changes capable of compromising the device's performance and/or resulting in tissue degeneration around the implanted electrode [15]. For this reason, laboratories around the world have tested different materials and designs for microwire arrays in rodents [16], [17], non-human primates [14], [18], [19] and humans [20].

However, despite the increasing popularity of chronic microelectrode implants, relatively little is known about their long-term effects on brain parenchyma. One of the initial events which is better understood is activation of microglial cells after the implant [18]. Microglial cells are very sensitive to early pathological conditions, even if they mean only variations in extracellular ionic concentrations [21]. These cells are fundamental for the control of brain homeostasis and integrate the intrinsic brain defense system [22], being immediately activated in several pathological conditions such as stroke, chemical and traumatic injuries and neurodegenerative disorders [21], [23]–[27]. The local activation of glial cells due to chronic electrode implants usually causes electrode encapsulation [28], and the resulting increase in electrical impedance of the recording tip over time [29]. This process presumably leads to an initial improvement of extracellular signal's selectivity, followed by a progressive decrease in the number of recorded neurons, until the complete cessation of signal [29].

Astrocytes are the major components of the encapsulating tissue that is deposited around chronically implanted electrodes [30]. These cells are usually recruited to the site of CNS injury, contributing to the glial scar that prevents axonal regeneration [31]. Some authors have proposed that glial encapsulation, a phenomenon called gliosis (i.e. a proliferation of astrocytes in CNS injured areas) [32], insulates implanted electrodes from nearby neurons, thereby hindering diffusion and increasing impedance [28] or creating an inhibitory environment for neurite extension.

Understanding the complex interactions between chronic neural implants and the cells that form the brain parenchyma is, therefore, a crucial step in developing stable, long-term microelectrodes. However, many questions still remain to be clarified, mainly regarding the effects of chronic multielectrode implants in tissue metabolism, cell structure, brain cytoarchitecture, and cell death, and how these potential changes relate to quality of electrophysiological recordings over time. To address these questions, we set out to evaluate how tungsten microelectrode arrays implanted in the rat brain affect biological markers of tissue metabolism, inflammatory response, immediate early gene (IEG) expression, cytoskeletal integrity, and apoptotic cell death. To provide a comprehensive picture of the effect of microelectrode implants in cortical homeostasis, we also investigated the correlation of these markers with electrophysiological signals over time. We choose the motor cortex as target of our study because of the relevance of this region to neuroprosthetic applications [9], [33], [34].

## Results

### Electrophysiology

**Figure 1:**
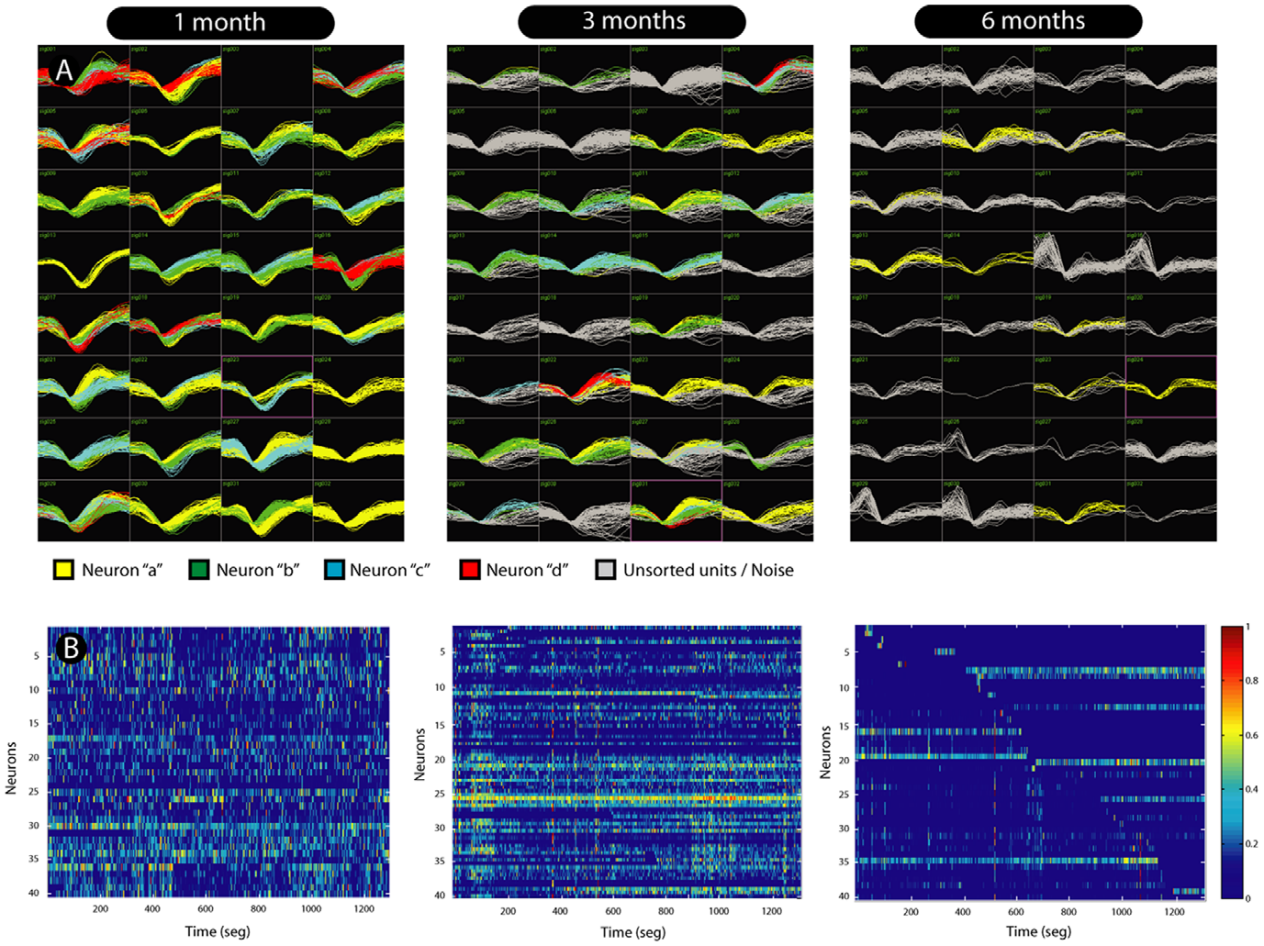
Recording session in a rat implanted during six months. Waveforms of 72 sorted units recorded with a 32 microwire array implanted in the motor cortex. Distinct neuronal units are well identified six months after implant (A). Neuronal activity varies with implantation time. In (B) we see representative examples of neuronal ensemble firing over implantation time, showing a natural decrease of firing rates and in the number of recorded neurons 6 months after array implantation.

**Figure 2:**
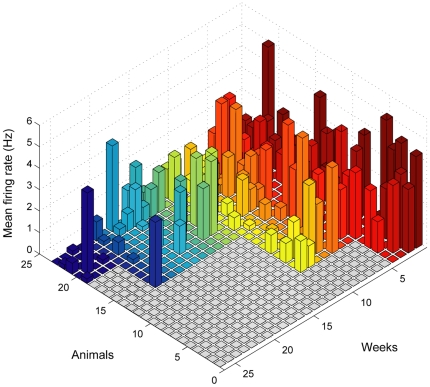
Neuronal recording average neurons/firing rate along weeks. Average number of neurons recorded over post-implantation time. It is possible to detect good electrophysiological signal 6 months after implantation. Progression of the time of recording (in weeks) is evidenced by the color code (first weeks: ‘hot’ colors; last weeks: ‘cold’ colors).

On average, 72±21 (mean±SD) neuronal units per session were chronically recorded in each animal. Recording sessions initiated between 7 and 14 days after the implant. Figure 1 illustrates the results from a recording session in a rat implanted during 6 months. Waveforms from 72 neuronal units recorded 1 month after array implantation are illustrated in the top left panel of Figure 1A, with a decay of around 45% of units recorded after 3 months (middle panel) and around 85% after 6 months (right panel) (1 month: 72±21; 3 months: 39±11; 6 months: 11±3.2; mean±SD). The bottom panels (B) show representative samples of neuronal ensemble firing over time, showing a decrease of firing rates and of the number of recorded neurons 6 months after array implantation. A summary of the total number of recorded neurons across animals shows the existence of single-unit activity even 6 months after implantation (Figure 2).

Concerning the analysis of LFP power, although LFP can be used to detect changes in recording quality over time, the LFP signal is very robust and changes little over months. In fact, it is very common that one can still record LFP from a given channel even after no spike signal can be detected. To assess this issue we did an analysis of power spectrum for different bands of frequency as function of time for channels with and without spikes. We first separated the LFP signal in standard spectral bands (0–4; 4–8; 8–12; 12–24, and 24–60 Hz). For each rat, the band power was divided by the total LFP power. Channels with spikes present were compared to channels without spikes at different time points. The results show that the relative LFP power in different bands varies similarly in both groups of channels (Figure S1).

### General histological pattern of implanted tissue

**Figure 3:**
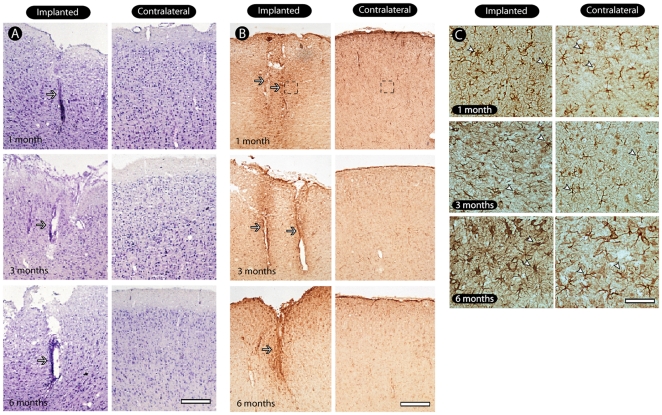
General pattern of tissue preservation in implanted sites. Nissl staining reveals a minimal cell loss in the implanted tissue (arrows), with a preservation of neurons near the electrode track and the absence of vacuolization and pycnotic profiles. In (B) we see GFAP immunostaining revealing astrocytosis restricted to the vicinity of electrode tracks, especially in later survival times, as a dense strip of cells throughout the implanted tissue. Notice the reactivity along electrode tracks (arrows). Astrocytes displayed a non-activated morphology with cells presenting non-hypertrophic cell bodies and absence of short and thick processes in contralateral hemisphere (C, right side, arrowheads). In the implantation site we identified an astrocytic activation, as reflected by the presence of cells displaying hypertrophic cell bodies and shorter and thicker processes (C, left side, arrowheads). Scale bars: 500 µm (A, B); 100 µm (C).

Figure 3A shows the typical pattern of electrode tracks left by the implants in sections obtained from animals at every survival time evaluated. The Nissl staining showed minimal cell loss in the implanted tissue, with a preservation of neuronal bodies near the electrode track and the absence of vacuolization. Pycnotic nuclei, an indirect evidence of cell death, were not detected. Based solely on the Nissl staining, the electrode arrays appear to be well integrated and tolerated for up to 6 months after implantation.

### Metabolic markers

**Figure 4:**
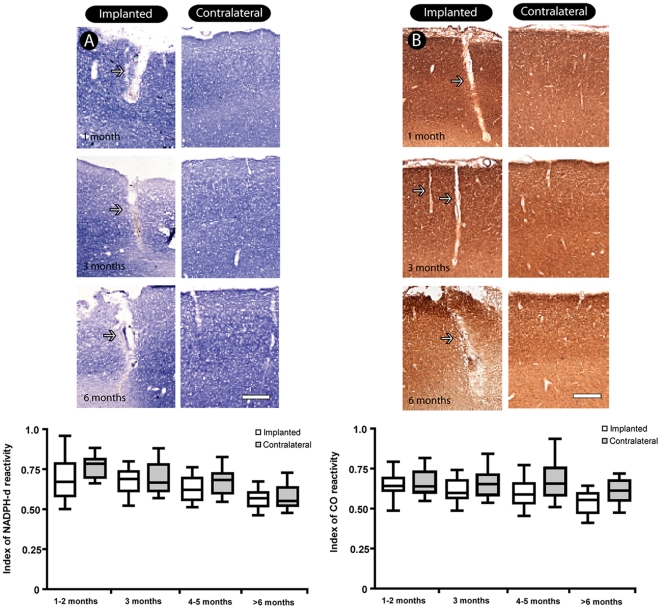
General pattern of histochemical markers in the implanted tissue. A small decrease is observed only in the implantation site (arrows), with a good pattern of preservation in the subjacent tissue for both techniques (NADPH diaphorase, A; cytochrome oxidase, B), as attested by densitometric analysis of the implanted regions when compared with their counterparts of the contralateral hemisphere (bottom of the figures). Values referred as mean±SEM. Scale bar in A: 500 µm.

Histochemistry for both NADPH-d and CO revealed a similar pattern of tissue preservation (Figure 4A,B, top). Decreased reactivity was restricted to tissue adjacent to the recording wires. Tissue located far from the immediate vicinity of the electrodes was not affected, showing normal reactivity, similar to the unaffected contralateral hemisphere. This qualitative pattern was confirmed by densitometric analysis: there was no significant difference between regions located in the vicinity of the implanted sites and their contralateral counterpart (Mann-Whitney test, p>0.05) (Figure 4A,B, bottom). We noticed a non-significant trend for a small reduction in reactivity in late survival times (Kruskal-Wallis - Bonferroni *post hoc* test, p>0.05).

### Pattern of neuronal labeling

**Figure 5:**
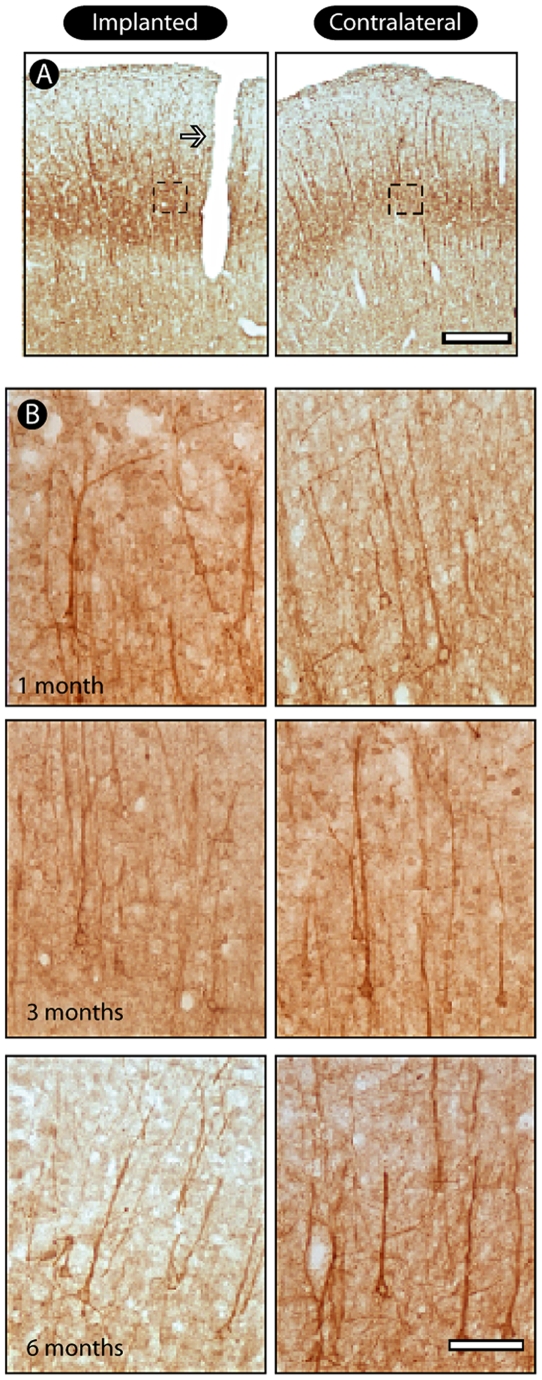
Pattern of neuronal labeling around electrode tracks. Using immunohistochemistry for NF-M we observed normal neuronal morphology around the electrode tracks (arrow) in all survival time groups it is possible to visualize a well-preserved pattern of both apical and some basal dendrites, as well as distinct pyramidal cell bodies (enlargements). Scale bars: 500 µm; 100 µm (enlargements).

NF-M, a marker of neuron integrity, revealed the presence of morphologically normal neurons around the electrode tracks in all survival times (Figure 5). Overall, it was possible to visualize a well-preserved pattern of both apical and basal dendrites, and unequivocally distinct pyramidal cell bodies in both implanted and contralateral hemispheres. In addition, we did not detect any difference in the pattern of cell labeling (Figure 5).

### Glial activation and inflammatory response around electrode tracks

Astrocytosis was observed around the electrode tracks, especially in later survival times, as a dense strip of cells throughout the implanted tissue (Figure 3B). In the implantation site we identified an astrocytic activation, as reflected by the presence of cells displaying hypertrophic cell bodies and shorter and thicker processes (Figure 3C). In the contralateral hemisphere astrocytes had a non-activated morphology with cells presenting non-hypertrophic cell bodies and absence of short and thick processes.

**Figure 6:**
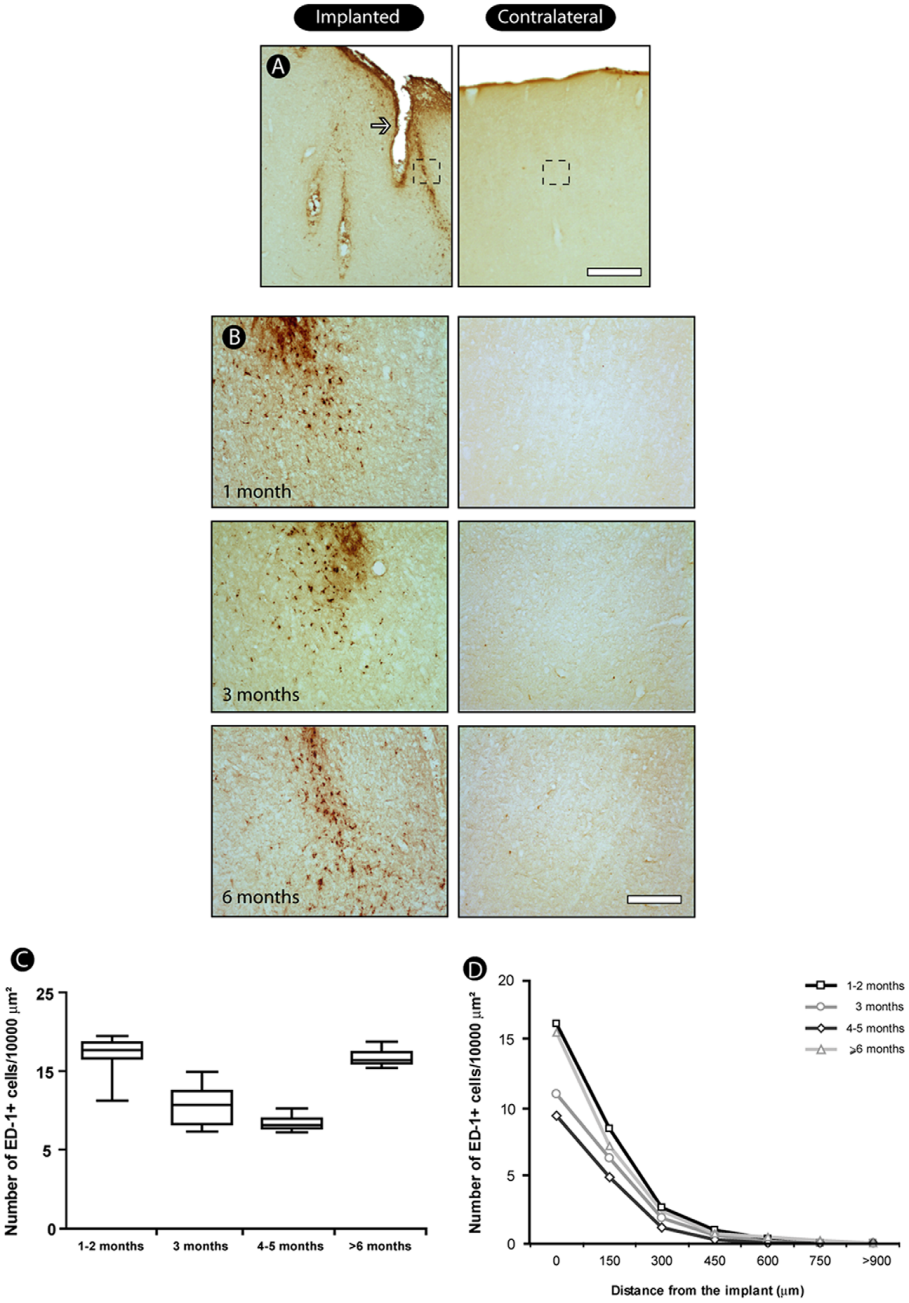
General pattern of inflammatory response. We found a relatively small but persistent and localized microglial activation consisting of macrophages/microglia located only nearly implantation site (A, arrow). Notice the similar morphological aspect of activated microglia in all groups (B). The quantitative analysis revealed a non-significant difference among groups, although there is a trend to a high number of activated microglial cells in the 6 months group (C). Quantitative analysis reveals that microglial activation is restricted to the vicinity of implants (D). Values expressed as mean±SEM. Scale bar: 500 µm; 100 µm (enlargements).

To evaluate the presence of activated microglia, we immunostained brain sections with ED-1. Glial reactivity to the microelectrode presence in all survival times was characterized by relatively small and localized cell activation, consisting of macrophages/microglia located only near the implantation site (Figure 6, top). Quantitatively, there was a small number of ED1-positive cells close to the microelectrode (Figure 6, bottom), with no significant difference among post-implantation times (p = 0.21, Kruskal-Wallis – Bonferroni *post hoc* test, corrected for the number of comparisons). We observed an absence of ED-1-reactive cells in a short distance away from the implanted sites and also in the contralateral hemisphere, used as an intrinsic control (Figure 6).

### Apoptotic profiles

**Figure 7:**
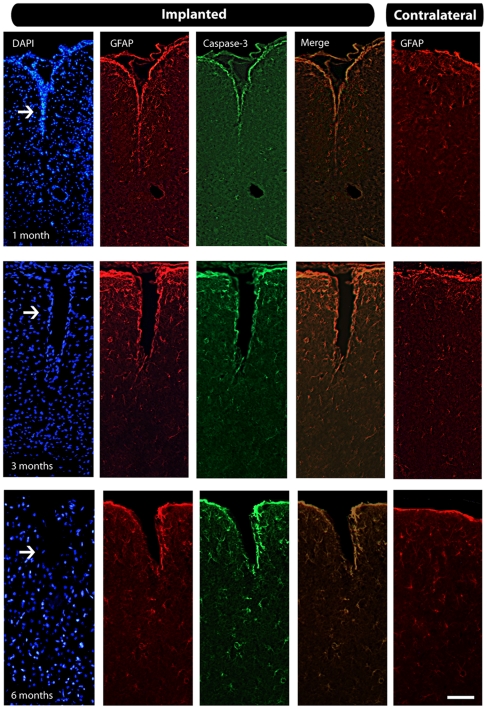
Relationship between cell death and astrocytosis in implanted tissue. We found a small amount of apoptotic cell death across groups located throughout the electrode tracks (arrows) even in the later group, as revealed by immunofluorescence. In addition we can see the astrocytes located around the electrode tracks in all time points. Scale bar: 500 µm.

We evaluated the presence of apoptotic profiles around electrode tracks using the caspase-3 immunohistochemistry. We found only a small amount of apoptotic cell death along the electrode track across survival groups (Figure 7), but never far from it, even in the 6-month survival group (Figure 7). In addition, since double-labeling using caspase-3 and GFAP antibodies revealed a strict co-localization, we propose that the observed small amount of cell death does not correspond to neuronal death. Also, as aforesaid, we did not detect pycnotic profiles using Nissl staining, corroborating the relative absence of apoptotic death.

### Immediate early-gene expression

**Figure 8:**
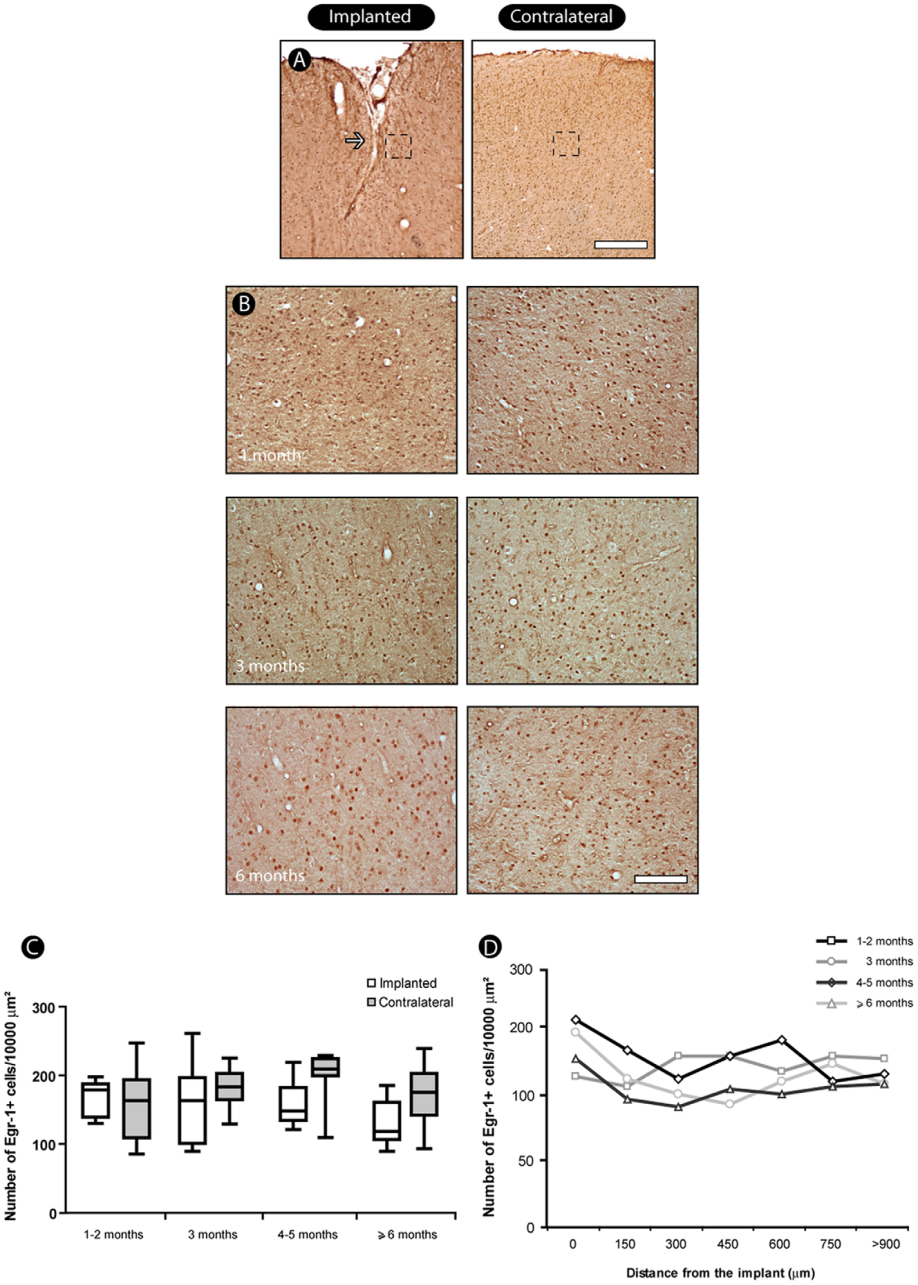
Immediate-early gene activation across implanted sites. We identified a normal pattern of labeling of Egr-1 protein, with a pattern of nuclear localization similar in contralateral and implanted hemispheres (A), with profiles varying from intensely to weakly reactive (B). There was no significant difference between implanted and contralateral regions until 6 months of electrode implantation (C). Quantitative analysis reveals that number of Egr-1-active cells does not differ significantly when regions near and far from the implant are compared (D). Values expressed as mean±SEM. Scale bars: 500 µm; 100 µm (enlargements).

The pattern of *egr-1* nuclear localization was similar in both contralateral and implanted hemispheres, with profiles varying from intensely to weakly reactive in every region (Figure 8). This pattern was confirmed quantitatively: there was no significant difference between implanted and contralateral regions until 6 months of electrode implantation (Figure 8).

### Relationship between histological pattern and electrophysiological signal

**Figure 9:**
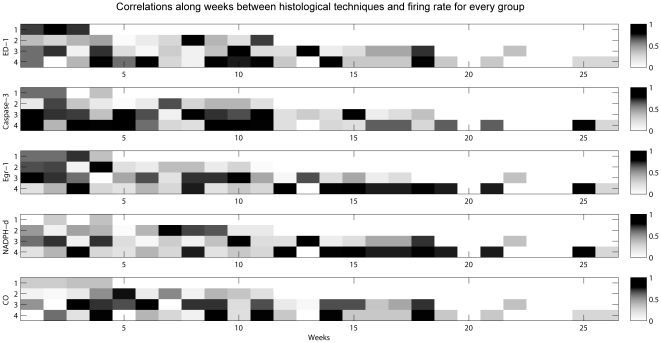
Correlation between different markers of tissue integrity and firing rates. In all groups histological markers exhibit higher correlations with firing rates during the initial post-implantation weeks, with a gentle decrease over time. The later group (up to 6 months) presented an increased correlation at the middle of implant period, mainly concerning histochemistry techniques. In addition, the early group (1–2 months) presented a relative constant correlation throughout the implantation period.

In order to relate the pattern of tissue integrity to the dynamics of electrophysiological changes, we calculated the non-parametric Spearman correlation coefficient between firing rate and several histological parameters, followed by a two-tailed *t* test for significance (Figure 9). Correlations were separately calculated for each group of implants and were based on median firing rates. For rate calculation, we divided the spike count of each neuron by the total time of recording. The resulting number allowed us to estimate the variation of spiking activity throughout the several weeks of recording (the absolute values are shown in Figure 2). The amount of cells positive for Egr-1 exhibit higher correlation values during the first weeks for all groups, with a slight decrease over time (Figure 9). The late group (up to 6 months) presented increased correlations in the middle of the implant period. In addition, the early group (1–2 months) presented a relative constant correlation throughout the implantation period. The same can be noticed when we correlated firing rates with the histochemical patterns of NADPH-d and CO. ED-1 and caspase-3 positive cells showed an increasing correlation with firing rates during the latter implantation period, well-correlated with the decrease of neuronal signal.

## Discussion

Here we have comprehensively characterized the general pattern of tissue reaction and tolerance to chronic cortical implants of tungsten microelectrode arrays, using a broad range of histological, histochemical and immunohistochemical methods. In addition, we established correlations between these markers and the quality of electrophysiological signals recorded by the arrays. We have obtained four main findings. First, the implanted tissue is histologically, structurally, and metabolically well-preserved. Second, electrode implantation does not affect the normal physiology of the implanted tissue, as indicated by IEG reactivity. Third, despite a small amount of inflammatory response and gliosis in the implanted sites, cell death is minimal after the multielectrode implantation. Finally, we have also observed that electrophysiological signals were still detectable even after 6 months of implantation, in correspondence with the general pattern of tissue integrity. There was, however, a significant decay in the number of recorded neurons over time.

### Chronic implants and tissue integrity

Biocompatibility can be defined as the capability of an implanted prosthesis to coexist in harmony with the host tissue without causing deleterious changes. Different materials have been used to manufacture biocompatible multielectrode arrays so far, such as microwire arrays and bundles [35], silicon [30] and platinum [18], with varied impacts on tissue integrity, as measured by the amount of reactive glia attracted to the implant site and by the decrease in the signal-to-noise ratio (SNR) of the electrophysiological signal. Other factors in addition to the electrodes' material should be considered in order to generate biocompatible and stable implants, such as electrode shape, size, texture, tip geometry and array configuration. Szarowski et al. (2003), in a comparative study evaluating the tissue response to silicon implants of different sizes, surface characteristics, and implantation procedures, reported that glial scar formation was unaffected by that range of variables, although distinct array geometries may influence the initial wound response [36]. In that work, however, the authors used only a small number of animals by group (1–2 animals), and therefore may have overlooked the inter-animal response variability. Ward et al. (2009) assessed the effectiveness of some commercial microelectrodes with distinct configurations [37]. According to these authors, none of the examined arrays can be considered superior to others, especially with respect to the relationship between electrophysiological signal and inflammatory response induced by the implant, although they did not quantify in detail the glial activation levels. Biran et al. (2005) evaluated the impact of the chronic silicon electrode arrays in the nervous tissue, observing an elevation of glial cells numbers (both astrocytes and microglia) accompanied by a decrease in the number of neurons in the electrodes' vicinity [38]. The development of this histological pattern was accompanied by a steady decrease in the SNR, suggesting that the glial response and cell loss impair single-unit recordings over time.

In the present work we observed a small degree of tissue alteration induced by the presence of tungsten microelectrode arrays. Tungsten is a good choice of material for chronic implants since it is a stable and inert metal, and has been successfully used to simultaneously record neuronal populations for several months and even years [3], [19], [35], [39]. In agreement with this, we detected small metabolic loss restricted to the sites of electrode implantation, a well-preserved structural organization, as revealed by the normal morphology of pyramidal cells, and a normal tissue physiology, indicated by unaltered levels of Egr-1 expression, directly involved with synaptic plasticity [40], [41]. In addition, neuronal death was not more prominent in the implanted sites. Altogether, these results indicate that Teflon-coated tungsten microwire arrays are well-tolerated by the nervous tissue. Some other factors that could explain these findings are the flexibility of the electrode's shaft and the shape of the tip. Tungsten microwires are sufficiently rigid to allow tissue penetration, and yet are flexible enough to prevent shear and tear due to their relative movement with respect to the brain. Biocompatibility is also aided by the use of blunt electrode tips, instead of electrodes with sharp tips that easily damage the tissue when movement occurs. The combination of these features likely explains why multielectrode arrays made of blunt tungsten wires may be chronically implanted for several months and still be sturdily tolerated by the nervous tissue, without causing widely deleterious effects.

In our study we observed the decay of the neuronal activity during implantation time. Based in the well-preserved pattern of neuronal morphology and Egr-1 expression, we believe that the progressive drop of neuronal activity is associated more directly to the glial encapsulation of the electrode rather than a loss of tissue functionality around it, a result well-correlated with previous reports (see [42] for a review). Further studies, mainly employing electronic microscopy to evaluate the tissue attached to the electrode after withdrawal from the parenchyma can help to clarify this issue.

Specifically concerning Egr-1 expression, since we did not apply extra sensory stimulation to the animals other than the normal amount of stimulation of being awake in a cage, we did not assess Egr-1 response to stimulation. Instead, we evaluated baseline levels of its expression, which tend to be high [43], [44] and proportional to neural activity [43], [74]. So, the fact that Egr-1 labeling was constant over time indicates that the decaying firing rates detected by the multielectrode implants in our study does not reflect a physiological decay, but rather an effect of gliosis.

### Electrode implants and inflammatory response

The inflammatory response is a very important physiological mechanism to safeguard tissue against the action of aggressive agents or mechanical/chemical injuries. Accordingly, no matter the array configuration and the material, the inflammatory response is always present in chronic implants and consequently electrodes tend to lose their effectiveness over time. The induction of tissue inflammation by chronic neural implants cannot be completely avoided because even a tiny mechanic lesion provokes alterations in the tissue milieu and a consequent recruitment of inflammatory cells and the glial activation [27]. Since it seems obvious that some inflammatory response will occur, especially during the process of electrode implantation, one of the main goals would be to ensure that the tissue reaction to the foreign body can be maintained at a tolerable level. Bjornsson et al. (2006) reported extensive short-term effects of electrode insertion, including cell death (both neuronal and glial), severed neuronal processes and blood vessels, mechanical tissue compression, and collection of debris resulting from cell death [45]. A prolonged and exacerbated inflammatory response mediated by pro-inflammatory cytokines such as tumor necrosis factor alpha (TNF-α), interleukin 1 beta (IL-1β), and nitric oxide (NO) can be highly harmful to the nervous tissue [46]–[48]. For instance, even when the foreign body cannot be degraded, as in the case with implanted electrodes whose material composition is resistant to enzymatic dissolution, yet the inflammatory reaction contributes to recording failure, by releasing necrotic substances into the immediate vicinity [49] and contributing to cell death around the electrode. However, the deleterious effects of inflammation can be modulated with pharmacological approaches, such as the administration of the N-methyl-d-aspartate receptor antagonist MK-801 and minocycline, a synthetic analogue of tetracycline [50]–[53], which can easily cross the blood-brain barrier [54], [55]. These drugs could act directly in the implantation site in order to control the inflammatory response, helping to sustain an effective electrophysiological record for a longer period of time.

It is important to keep in mind regarding tissue response to microelectrode implants that although microglial cells seem to have the same role as resident macrophages in the nervous system of all mammalian species, other functions could be more species specific [56]. In this context, it is interesting to have in perspective the action of these cells in humans submitted to a chronic multielectrode implant. For example, in rats, reactive microglia produces a higher amount of nitric oxide (NO) than in humans [56]. This interspecies difference could explain, for instance, why chronic recordings in non-human primates last longer than in rats. Although we cannot generalize the results we found in rats, our findings are promising, given the lesser reactivity of human microglial cells as compared to the rat's [56]. The use of anti-inflammatory substances could help maintain the implant's functionality for a longer time by the selective blockade of microglial activity, since reactive oxygen species produced by microglia are widely harmful to the nervous tissue [47].

### Conclusion

Our results have particular relevance for the future development of cortical neuroprosthetic devices [6], [9], [11], [14], [57]. The main long-term goal of this research line is to contribute with information that may in the future allow neurological patients to use spared brain tissue to compensate for the loss of function, based on the correlation between neuronal activity and specific behaviors [58]. To this end, more studies need to be carried out, testing for instance alternative electrode array configurations, in order to provide a stable and biocompatible interface for humans patients suffering degenerative diseases, such as Parkinson's, Alzheimer's, cerebral palsy, amyotrophic lateral sclerosis, stroke and spinal cord injury [33], [59]–[62]. Further studies employing the use of specific substances to control the inflammatory process triggered mainly during the electrode implant can also contribute to increase the effectiveness of the implant.

## Materials and Methods

### Multielectrode implants

Twenty-four adult male Wistar rats (300–350 g) were used in this study. All experimental procedures were strictly in accordance with the National Institute of Health Guide for the Care and Use of Laboratory Animals (NIH Publications No. 80–23) and were approved by the Edmond and Lily Safra International Institute of Neuroscience of Natal Committee for Ethics in Animal Experimentation (permit ID # 04/2009).

**Figure 10:**
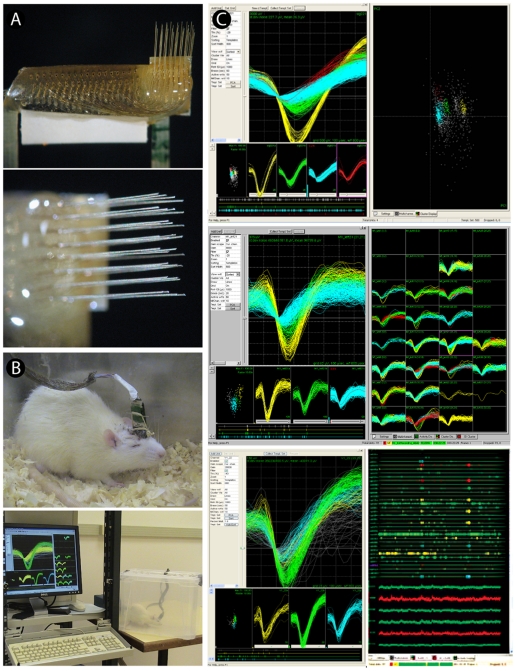
General aspect of multielectrode array and electrophysiological signal recording. A 32 channel microelectrode array (A). In (B) we see a recording session after a 1-month of implantation procedure, with the headstage connected to the rat. (C) shows examples of sorted waveforms using the Real-time Acquisition System Programs for Unit Timing in Neuroscience (RASPUTIN - Plexon Neurotechnology Research Systems) after one (top), three (middle) and six months of implantation (bottom). Samples of channels in which at least three clearly well-isolated single neurons are identified and recorded in real-time during the exploratory activity of the animal.

Surgeries for multielectrode implantation were performed in rats deeply anesthetized with 100 mg/kg of ketamine chlorhydrate and 5 mg/kg xylazine chlorhydrate, as described in detail elsewhere [29]. Briefly, rats were placed in a stereotaxic head holder, and a small craniotomy was made over the implant target area, the primary motor cortex, using the following coordinates (in millimeters relative to bregma): 1.0–3.0, anteroposterior (AP); 2.0–3.0, mediolateral (ML); 1.8–2.0, dorsoventral (DV) [63]. Each animal was slowly implanted with multielectrode arrays (4×8 with 500 µm spacing) made of 32 Teflon-coated tungsten electrodes (35-µm microwire diameter, 1.5 MOhm at 1.0 KHz), attached to an Omnetics connector (Omnetics Connector Corp., USA) (Figure 10). All the implantation procedures were guided by multiunit recordings, though without online sorting, for a better dorsoventral localization. Although this procedure prolongs the surgery, it ensures best results for chronic spike recordings. In our surgery procedure the connector of the electrode array always stayed above the cranium, outside the tissue, in order to prevent any alteration in the cortical mantle (Figure S2).

After 1 week to surgical recovery, animals underwent weekly sessions of electrophysiological recordings. Four survival time groups were formed, according to the time of killing: 1–2, 3, 4–5 and ≥6 months after implant.

### Electrophysiological recordings

A 32-channel multi-neuron acquisition processor (MAP, Plexon Inc., USA) was used for digital spike waveform discrimination and storage. Single-unit recording sessions lasted for at least 30 minutes in a weekly basis, while the rats moved freely in their cages (Figure 10). Online spike sorting was conducted with the help of the SortClient 2002 software (Plexon Inc., USA). All the action potentials of a maximum of 4 neuronal units per channel were sorted online by only one investigator and validated by offline analysis (Offline Sorter 2.3, Plexon Inc., USA) according to the following cumulative criteria: a) voltage thresholds >3 standard deviations of amplitude distributions; b) signal-to-noise ratio >2.5 (as verified on the oscilloscope screen); c) less than 0.5% of inter-spike intervals (ISI) smaller than 1.0 ms; d) stereotypy of waveform shapes, as determined by a waveform template algorithm and principal component analysis. Local field potentials (LFP) were simultaneously recorded from the wires and pre-amplified (500x), filtered (0.3–400 Hz), and digitized at 500 Hz using a Digital Acquisition board (National Instruments, USA) and a MAP box (Plexon Inc., USA) (Figure 10).

**Figure 11:**
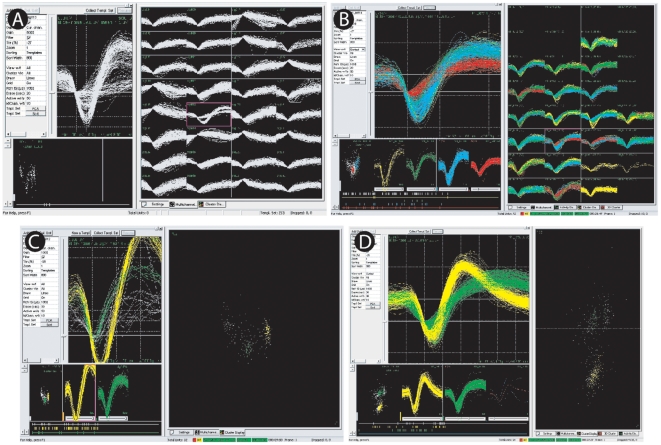
Multielectrode recording. All the electrode implants were guided by multiunit recordings, though without online sorting (detection by threshold or classification) (A). Multiunit activity in a single channel. The default threshold for the highlighted channel allowed the detection of multiple spikes and three units could have been easily isolated (A, left side). After adequate thresholding it was possible to isolate distinct units in the channel (defined by different colors) (B). Large amplitude waveforms stand out from those closer to threshold (C). PCA analysis was used to single out three different units in this channel (D, right side).

Sort Client, the main user interface in the recording software from Plexon Inc. (RASPUTIN software), allows spike sorting through two basic tasks: detection and classification. A voltage-threshold trigger is one of the simplest ways to detect spikes and sorting them in real time according to their shape. The detection process can be easily applied within a brief period of time for all 32 channels, and the classification of neurons was made individually, channel by channel. Usually large amplitude waveforms were quite different from those closer to threshold, allowing an unambiguous discerning of distinct neurons (Figure 11).

We compared sorting performed both online and offline for consistency. The electrophysiological sorting criteria were updated in every recording session, but we used the manual online sorting capability of the SortClient software instead of the automated online sorting. The selection of individual neurons in all recording sessions followed the same criteria over time, as defined above. In this way, we could follow the amount of clearly distinct individual neurons, despite variations in the background noise.

### Perfusion

At the end of pre-specified survival times, animals were deeply anesthetized with 5% isoflurane and overdosed with sodium thiopental (90 mg/kg). Animals were then perfused intracardially with 0.9% heparinized saline followed by 4% paraformaldehyde in 0.1 M phosphate buffer (PB), pH 7.4. Next, the brains were removed from the skull and immersed in 20% sucrose in 0.1 M phosphate buffer saline (PBS) for 12 h. The tissue was then frozen in a embedding medium (Tissue Tek, Sakura Finetek, Japan) and sectioned frontally at 20 µm in a cryostat (Carl Zeiss Micron HM 550, Germany). The sections were mounted on electrically charged glasses (Super Frost Plus – VWR International, USA) and submitted to histological, histochemical and immunohistochemical procedures.

### Basic histology and histochemistry

To evaluate tissue reactivity around the electrode tracks we used nicotinamide adenine dinucleotide phosphate-diaphorase (NADPH-d) and cytochrome oxidase (CO) histochemistries, both outstanding markers of cortical modules and layers [64], [65]. In brief, to reveal NADPH-d activity, brain sections were washed in 0.1 M Tris buffer, pH 8.0 and incubated in a solution containing 0.6% malic acid, 0.03% nitroblue tetrazolium, 1% dimethylsulfoxide, 0.03% manganese chloride, 0.5% β-NADP and 1.5% Triton X-100 in 0.1 M Tris buffer, pH 8.0 [66]. The histochemical reaction was monitored every 30 min to avoid overstaining and was interrupted by rinsing sections in 0.1 M PB. We revealed CO activity by incubating sections in a solution containing 0.05% diaminobenzidine (DAB), 0.03% cytochrome *c* and 0.02% catalase in 0.1 M PB [67]. Similar to NADPH-d, the CO histochemical reaction was monitored every 30 minutes and was terminated by washing sections in 0.1 M PB.

Alternate sections were stained with cresyl violet (Nissl method) in order to identify the location of the electrode tracks. Before visualization, all sections were dehydrated and coverslipped with Entellan (Merck, Germany).

### Immunohistochemistry

In order to evaluate tissue integrity, we performed a series of immunohistochemical procedures. We labeled activated microglia with ED-1 antibody (1∶500; Serotec, UK) [68]. Astrocytes were labeled with an antibody against the glial fibrillary acid protein (GFAP; 1∶500; Sigma Company, USA) [69]. IEG activation was measured with immunohistochemistry against *egr-1* (1∶100; Santa Cruz Biotechnology, USA), a well-known marker of calcium-dependent neuronal activity [70]. Neurofilament M (NF-M), a structural constituent of neuronal cytoskeleton, was revealed using a monoclonal NF-M antibody (1∶100; Santa Cruz Biotechnology, USA) [71]. Apoptotic cells were labeled using caspase-3 antibody (1∶250; Promega, USA), a very sensible marker of these cells in the brain [72]. In brief, the sections were washed during 20 minutes in phosphate buffer saline-Tween (PBS-T) and incubated in a blocking buffer solution (0.5% fresh skim milk and 0.3% Triton X-100 in 0.1 M PBS) for 30 minutes to block non-specific binding. Afterward, sections were incubated overnight in primary antibody (diluted in blocking buffer) at 18°C, washed in PBS-T by 20 minutes, incubated with a biotinylated secondary antibody (1∶200, Vector Labs, USA; diluted in blocking buffer) for 2 h, washed during 20 minutes in PBS-T, and then incubated in avidin-biotin-peroxidase solution (Vectastain Standard ABC kit, Vector Labs, USA) for 2 h. Slides were then placed in a solution containing 0.03% 3,3′ diaminobenzidine (DAB) (Sigma Company, USA) and 0.001% hydrogen peroxide in 0.1 M PB, dehydrated and coverslipped with Entellan (Merck). In order to certify the specificity of the labeling the primary antibodies were replaced by blocking buffer in some test sections.

To determine which cells were apoptotic, animals from every group had some sections immunohistochemically stained using fluorescent labels. Briefly, sections were washed during 1 h in 0.5% Triton X-100 in 0.1 M PB and pre-incubated in blocking buffer solution during 30 minutes. Thereafter, the sections were incubated overnight with primary antibodies (NF-M, GFAP and caspase-3) at 18°C. Sections were then washed in 0.1 M PB (3x, 5 minutes each) and incubated in a mixture of Alexa Fluor 488-conjugated horse anti-mouse and Alexa Fluor 594-conjugated goat anti-rabbit overnight (1∶500 in 0.1 M PBS, Invitrogen, USA). Finally, the sections were mounted using Vectashield mounting medium for fluorescence (DAPI/Antifade solution) (Vector Labs, USA) and sealed with Entellan (Merck). The following combinations were evaluated: NF-M with caspase-3 and GFAP with caspase-3.

### Qualitative and quantitative analysis

NADPH-d and CO reactivities were assessed by optical densitometry with the Image J software (http://rsb.info.nih.gov/ij/). Measurements were obtained inside a 0.02 mm^2^ square window positioned across electrode tracks. To minimize the effects of within-group variability, we adopted a normalized scale based on the reactivity of the underlying white matter (averaged over measurements of 10 different sites using the same window). For each animal, a contrast index was calculated according to the equation: C = (G–W)/(G+W) [73], in which G is the average optical density (OD) of cortical tissue around the electrode tracks, and W is the OD of the underlying white matter. Reactivity values were obtained for each implanted region and compared with their contralateral counterpart. Statistical significance was assessed using non-parametric Mann-Whitney test and Bonferroni *post hoc* test with an alpha level of 0.05.

To quantify the immunohistochemical data, we counted the total number of cells labeled by *egr-1*, ED-1 and caspase-3 in the region around electrode tracks in the four survival time groups (1–2, 3, 4–5 and ≥6 months of implant) using the *Neurolucida* system (MBF Bioscience Inc., USA). For every marker, we quantified 3 sections per animal, sampling tissue from regions where the electrode tracks could be unequivocally observed (*n* = 4–5 animals by group). The cell density (cells/10,000 µm^2^) was estimated using an automatic grid from the *Neurolucida* program. The contours of positive astrocytes and NF-M labeled cells were qualitatively evaluated across groups in order to characterize astrocytic activation and general morphology, respectively. The hemisphere contralateral to the implant was adopted as an intrinsic control in all animals. Average values for all measurements were assessed with non-parametric Kruskal-Wallis test and Bonferroni *post hoc* test. The criterion for statistical significance was preset at an alpha level of 0.05. Average values are expressed as mean±standard error of mean (SEM).

To obtain digital images we used a CX9000 camera (MBF Bioscience Inc., USA), attached to a light field Nikon Eclipse 80i optical microscope (Nikon, Japan - 10x and 20x objectives).

### Co-localization of NF-M and GFAP with caspase-3

Double-labeled sections for NF-M with caspase-3 and GFAP with caspase-3 were evaluated with the aid of a Nikon Eclipse 80i microscope (Nikon, Japan) equipped with a mercury fluorescence light source. Images were captured with the abovementioned digital camera at a single focal depth in the superficial portion of the section, to which both primary antibodies had penetrated. The images (20 and 40x objectives) were captured for each combination of antibodies from different sections processed. A composite image was obtained by superimposing the two images captured for each fluorophore using Image J software.

### Electrophysiological signal

We evaluated the quality of the electrophysiological signal by comparing the total number of neuronal units observed in the first and last recording sessions. We also characterized the decay of recorded units over time, thus assessing the quality of signal throughout all weeks of recording.

### Correlation between electrophysiological variables and histological features

In order to measure the relationship between the pattern of electrophysiological records throughout implantation and the tissue integrity, we calculated the non-parametric Spearman correlation coefficient of (or between) neuronal average firing rate (or average spiking counting), taken for each week, and distinct histochemical/immunohistochemical techniques, followed by a two-tailed *t* test for significance (with p<0.05) using Matlab 2008 software (The MathWorks Inc., USA).

## Supporting Information

Figure S1 **Relative LFP power across distinct frequency** **bands varies little over time, and does not differ between channels** **with or without spike signals.** The top panels depict raw LFP signals recorded at different time points (weeks), obtained from channels with or without spike signals (red and blue lines, respectively). The remaining panels show the temporal evolution of the relative LFP power within standard spectral bands, for the two groups of channels described above.
(TIF)

Figure S2 **Multielectrode implantation.** Figure showing the procedure of multielectrode implantation. In our surgeries the connector of the electrode array always stayed above the cranium, in order to prevent any alteration in the cortical mantle (zoomed at the right side).
(TIF)
