# Supplementary figures and images for: Comprehensive Analysis of Tissue Preservation and Recording Quality from Chronic Multielectrode Implants
Source: PLoS One. 2011 Nov 9;6(11):e27554. doi: 10.1371/journal.pone.0027554 (PMC3212580; doi:10.1371/journal.pone.0027554)

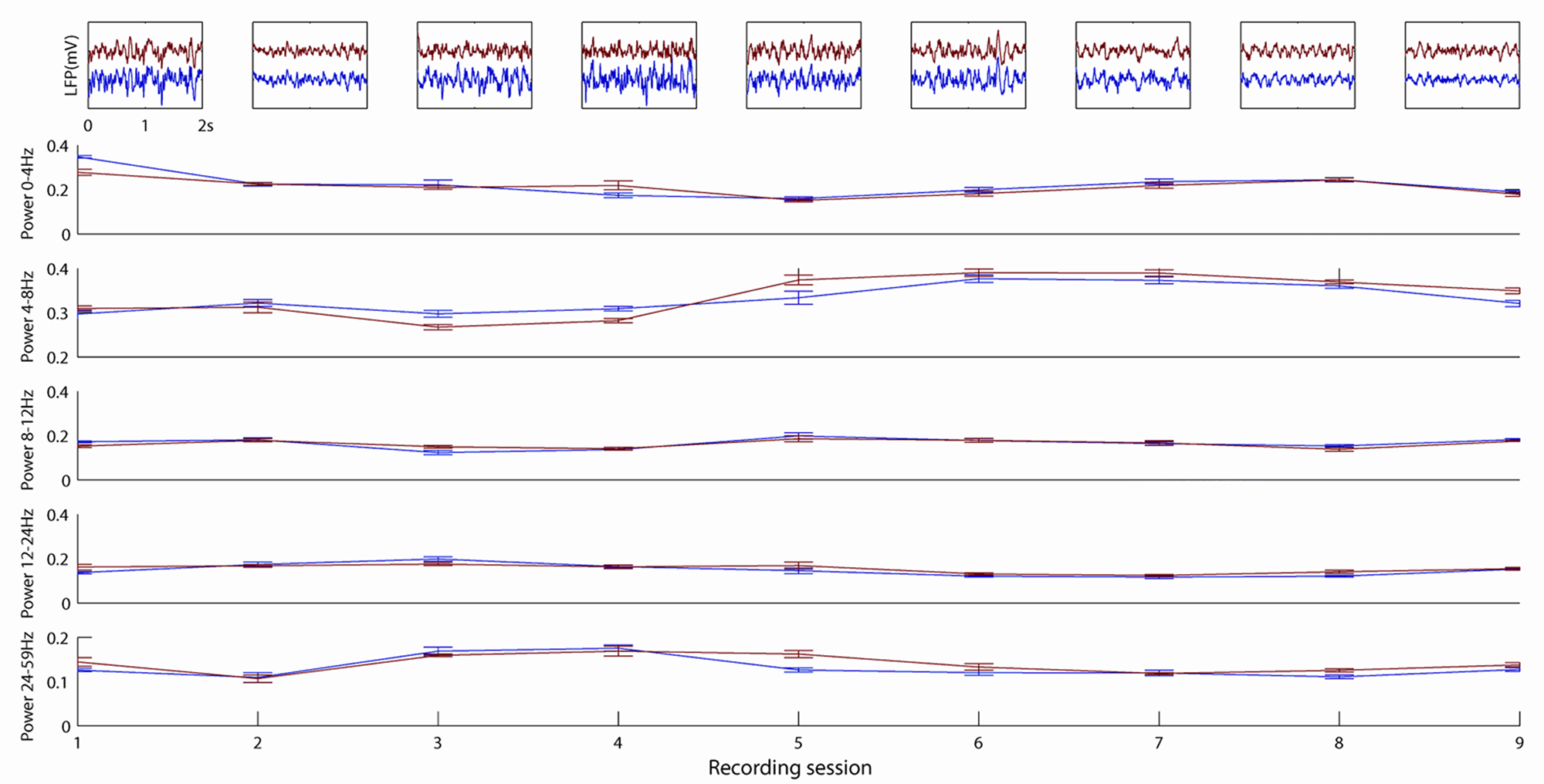

Supplement: Figure S1 — Relative LFP power across distinct frequency bands varies little over time, and does not differ between channels with or without spike signals. The top panels depict raw LFP signals recorded at different time points (weeks), obtained from channels with or without spike signals (red and blue lines, respectively). The remaining panels show the temporal evolution of the relative LFP power within standard spectral bands, for the two groups of channels described above. (TIF) [file pone.0027554.s001.tif]

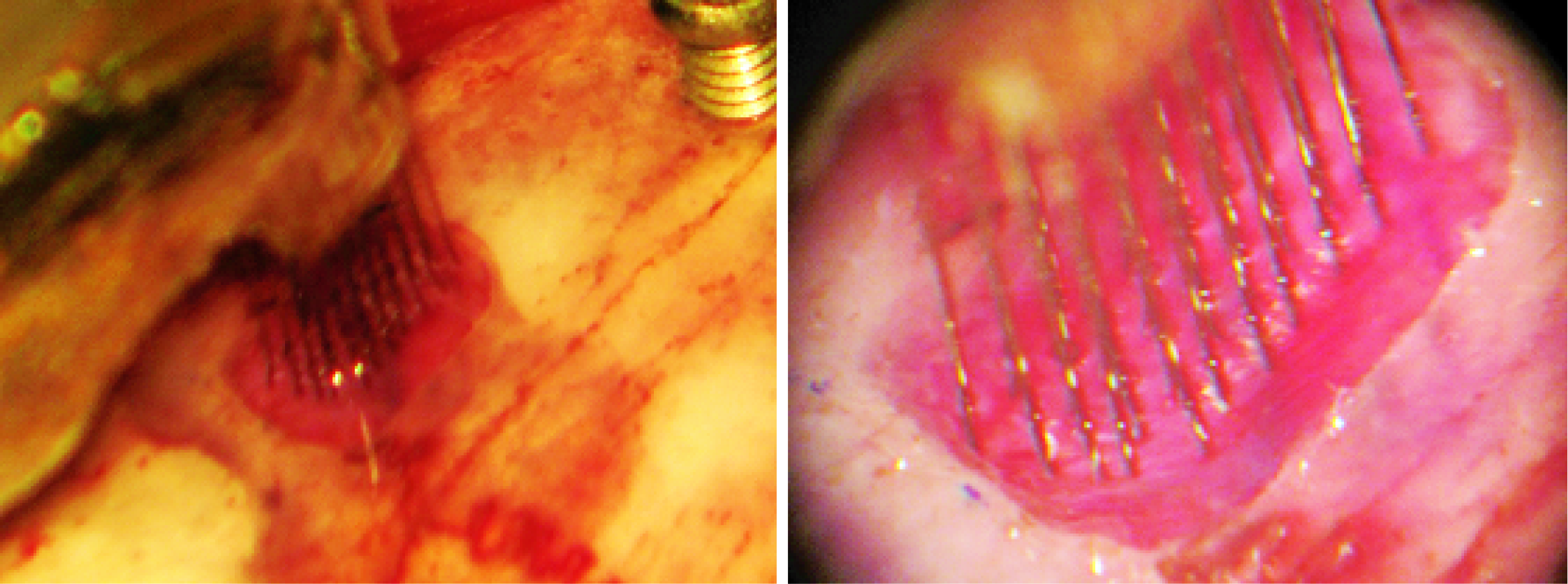

Supplement: Figure S2 — Multielectrode implantation. Figure showing the procedure of multielectrode implantation. In our surgeries the connector of the electrode array always stayed above the cranium, in order to prevent any alteration in the cortical mantle (zoomed at the right side). (TIF) [file pone.0027554.s002.tif]
